# Comment on: Matsumoto et al.

**DOI:** 10.1002/clt2.70000

**Published:** 2024-11-09

**Authors:** Molly Cremin, Sadhbh Hurley, Juan Trujillo

**Affiliations:** ^1^ Paediatric Department Cork University Hospital Cork Ireland; ^2^ Department of Paediatrics and Child Health University College Cork Cork Ireland

Dear Editor,

We would like to respond to the recent publication by Matsumoto et al. “Milk ladder versus early oral immunotherapy in infants with cow's milk protein (CMP) allergy” which we read with interest^1^ In this single center retrospective observational study the authors compared the efficacy and safety of milk ladder (ML) with early‐oral immunotherapy (E‐OIT) in children under the age of 2 years.

For over 10 years, milk ladders have been used as the mainstay of treatment for both IgE and non‐IgE mediated cow’s milk protein allergy (CMPA) in Ireland. As described in this publication, the ML involves the home‐based graded reintroduction of CMP containing foods in a stepwise fashion from least to most allergenic forms. This practice has been deemed safe and successful in Irish settings.^2^ As the use of Dietary advancement therapies (DATs) including MLs and OIT become more widespread in the global allergy community we strongly support the recollection of local data and thank the authors for publishing this paper which adds to the collective knowledge on the treatment of CMPA. This paper led us to consider our practice for patient selection and the differences between what is possible and practical in the clinical setting versus what is best practice.

In clinical practice the use of clinical history of parental reported immediate symptoms and proof of sensitisation is used to confirm milk allergy instead of the gold‐standard Oral Food Challenge (OFC). Prior to the initiation of OIT international guidelines indicates the use of OFC to confirm the diagnosis.^3^ In the protocol described by Matsumoto et al. they included patients with either parent reported immediate symptoms or proof of sensitisation (Milk specific IgE > 5 kUA/L). We agree with the authors that this is a limitation of the study. We feel that in the absence of a challenge proven allergy, it is likely that this cohort included children with parent reported symptoms alone (with no sensitisation or allergy) or asymptomatic sensitisation.

Previous studies comparing the use of OIT with total milk avoidance identified approximately half of children screened with parent reported symptoms AND evidence of sensitisation (IgE > 0.35) had a negative double‐blind placebo‐controlled food challenge to milk.^4^

The same limitations were discussed in research from our Irish team, we compared the use of ML and milk avoidance in real‐life diagnosed CMPA patients (positive clinical history and allergy tests without OFC) and decided to label the primary outcome as reintroduction of milk instead of tolerance.^5^

DATs can be used in high‐risk patients for the first introduction of CMP. This may be helpful when there is hesitation to introduce milk and can enable and empower families to introduce it at home in a graded way. For example, in high risk allergy patients that have no history of reaction or sensitisation to milk this practice for allergen introduction would be classified as Primary allergy prevention. In the case of patients milk sensitised with no history of reaction an introduction of milk using the ML could prevent the development of milk allergy and subsequently be classified as Secondary allergy prevention.^6^ In this study by Matsumoto et al, we wonder if some patients reported as tolerant following ML or E‐OIT could have instead undergone a primary or secondary CMPA prevention management.

This article made us re‐evaluate our own treatment outcomes and reflect that while DATs encompass different active treatments, in clinical practice we often do not differentiate between a graded re‐introduction and oral immune tolerance induction of an allergy.^6^

For us, this paper by Matsumoto et al. was an excellent study to remind us that without prior confirmation of allergy (with OFC) before commencing DAT or E‐OIT that we cannot label that this intervention targets an already allergic patient (tertiary prevention) and claim tolerance was achieved.^6^

## AUTHOR CONTRIBUTIONS

**Molly Cremin**: Writing—original draft; writing—review and editing. **Sadhbh Hurley**: Writing—original draft; writing—review and editing. **Juan Trujillo**: Writing—original draft; Writing—review and editing; supervision.

## CONFLICT OF INTEREST STATEMENT

The authors declare no conflicts of interest.

## Data Availability

Data sharing not applicable to this article as no datasets were generated or analysed during the current study.

## References

[1] Matsumoto Y , Fujita M , Ayumi T , Takamasu T , Inuo C . Milk ladder versus early oral immunotherapy in infants with cow’s milk protein allergy Clin Transl Allergy [Internet]. 2024;14(8). [cited 2024 Aug 14]. 10.1002/clt2.12388 PMC11309850 39117577

[2] d'Art YM , Forristal L , Byrne AM , et al. Single low‐dose exposure to cow's milk at diagnosis accelerates cow's milk allergic infants' progress on a milk ladder programme. Allergy. 2022;77(9):2760‐2769. 10.1111/all.15312 35403213 PMC9543429

[3] Santos A , Riggioni C , Ioana A , et al. EAACI guidelines on the diagnosis of IgE‐mediated food allergy. Allergy. 2023;78(12):3057‐3076. 10.1111/all.15902 37815205

[4] Lee JH , Kim WS , Kim H , Hahn YS . Increased cow’s milk protein–specific IgG4 levels after oral desensitization in 7‐ to 12‐month‐old infants. Ann Allergy Asthma Immunol. 2013;111(6):523‐528. 10.1016/j.anai.2013.09.001 24267363

[5] Trujillo J , Cronin C , Heng TA , et al. A retrospective comparison of IgE‐mediated cow’s milk protein allergy management strategies in pediatric cohorts. Pediatr Allergy Immunol. 2024;35(7). 10.1111/pai.14195 38989807

[6] Cronin C , Salzburg N , Woon Y , Wurttele JT . Primary, secondary and tertiary prevention of food allergy: current practices and future directions. Allergol Immunopathol. 2023;52(2):32‐44. 10.15586/aei.v52i2.1023 38459888

